# Draft Genome Sequences of Two Brazilian Cyanobacterial Strains of *Cylindrospermopsis raciborskii*: Differences in Membrane Transporters, Saxitoxin Production, and Antioxidant Activities

**DOI:** 10.1128/genomeA.00879-17

**Published:** 2017-10-26

**Authors:** Luísa Hoffmann, Ravi José Tristão Ramos, Iame Alves Guedes, Pamela Fernandes Costa, Catarina Rufino Diniz Miguel, Sandra Maria Feliciano de Oliveira e Azevedo, Rosane Silva

**Affiliations:** a Carlos Chagas Filho Institute of Biophysics, Federal University of Rio de Janeiro, Rio de Janeiro, Brazil; b Department of Biotechnology, Federal Institute of Education, Science and Technology of Rio de Janeiro, Rio de Janeiro, Brazil

## Abstract

We report here the draft genome sequences of two Brazilian strains of *Cylindrospermopsis raciborskii*, a saxitoxin-producer (CYRF) and a non-saxitoxin producer (CYLP), with each strain comprising one assembled scaffold. We revealed differences in the compositions of gene members coding for membrane transporters and antioxidant activities between the strains.

## GENOME ANNOUNCEMENT

*Cylindrospermopsis raciborskii* is a filamentous freshwater cyanobacterium initially isolated from a tropical site. This species was recently described in all continents and is classified as an invasive species (1). *C. raciborskii* strains can produce saxitoxin (STX), cylindrospermopsin (CYN), or neither toxin depending on their biogeographical distribution. Strains isolated from Australia, New Zealand, and Asia produce CYN, those from South America produce STX, and those from Europe are not toxic (1, 2).

We present here two strains of *C. raciborskii*, CYLP and CYRF, isolated from Brazilian freshwater ecosystems. CYLP was isolated from Paranoá Lake in the Federal District (15°49′S, 47°49′W) and does not produce STX or CYN. CYRF was isolated from the Funil Reservoir in Rio de Janeiro State (22°31′43″S 44°34′09″W) and is an STX producer (3). Both strains were grown in ASM-1 medium at 24 ± 1°C under a 12/12-h light/dark cycle, with a photosynthetic photon flux density of 50 µmol m^−2^ s^−1^. Cultures were collected at the exponential-growth phase, centrifuged at 3,500 × *g*, and washed twice with sterile ASM-1 medium. The remaining pellet was used for the DNA extraction. Genomic DNA (gDNA) was extracted using xantonato (4), quantified using a NanoDrop spectrophotometer (Thermo Scientific, USA), and mechanically fragmented using a sonicator Bioruptor UCD-200 (Diagenode, USA). Fragmentation quality was assessed using microfluidic electrophoresis (Bioanalyzer; Agilent Technologies, USA). Libraries for massively parallel sequencing were constructed using a 400-bp kit (Ion Xpress Plus gDNA fragment library; Thermo Scientific). Whole-genome sequencing was performed with an Ion PGM platform using an Ion 318 Chip (Thermo Scientific). After sequencing, the reads were extracted and trimmed by quality (Phred ≥ 20) and size (>35 nucleotides [nt]) using the CLC Genomics Workbench version 7.5 software (CLC bio, Qiagen, Denmark). *De novo* assembly of the trimmed reads was performed using MIRA Assembler version 4.0, and the resultant contigs were improved using a multidraft-based scaffolder (MeDuSa Web server) (5) using *C. raciborskii* CS-505 (NCBI reference sequences NZ_LYXA01000001 to NZ_LYXA01000006) as the reference genome. The final assemblies of CYLP and CYRF resulted in 188 and 168 contigs, total sizes of 3,972,140 and 4,150,301 bp, G+C contents of 40.09% and 40.18%, *N*_50_ values of 3,476,922 and 3,760,889 bp, *N*_90_ values of 12,112 and 3,760,889 bp, 353 and 353 subsystems, 5,250 and 5,038 coding sequences, and 54 and 62 predicted RNA genes, respectively. Annotation was performed using the Rapid Annotations using Subsystems Technology (RAST) 2.0 server (6) and Blast2GO. Secondary metabolites were assessed using the antiSMASH (Antibiotics & Secondary Metabolite Analysis SHell) software (7). The annotation revealed that CYRF has genes that are not present in CYLP for membrane transporters, such as those for sodium-driven multidrug and toxic compound extrusion proteins, the HlyD family secretion protein, major facilitator superfamily proteins, and toxin secretion ABC transporters, or for nonribosomal peptide synthetases, such as ketosynthase and hassalidin synthase (8). In contrast, CYLP has genes for antioxidant activity that were not present in CYRF. However, the two strains presented similar terpene and aryl-polyene synthase genes. These results indicate that the two Brazilian *C. raciborskii* isolates are different ecotypes.

### Accession number(s).

This whole-genome shotgun project has been deposited in DDBJ/ENA/GenBank under the accession no. NPIK00000000 for CYLP and NPIL00000000 for CYRF. The versions described in this paper are the first versions, NPIK01000000 and NPIL01000000.
